# A psychometric evaluation of the Gender Bias in Medical Education Scale

**DOI:** 10.1186/s12909-016-0774-2

**Published:** 2016-09-29

**Authors:** Rhiannon B. Parker, Philip D. Parker, Theresa Larkin, Jon Cockburn

**Affiliations:** 1 University of Wollongong, Northfields Ave, Wollongong, NSW 2522 Australia; 2 Institute of Positive Psychology and Education, Australian Catholic University, 25A Barker Road, Strathfield, Australia

**Keywords:** Gender, Medical education, Psychometrics

## Abstract

**Background:**

Gender bias within medical education is gaining increasing attention. However, valid and reliable measures are needed to adequately address and monitor this issue. This research conducts a psychometric evaluation of a short multidimensional scale that assesses medical students’ awareness of gender bias, beliefs that gender bias should be addressed, and experience of gender bias during medical education.

**Methods:**

Using students from the University of Wollongong, one pilot study and two empirical studies were conducted. The pilot study was used to scope the domain space (*n* = 28). This initial measure was extended to develop the Gender Bias in Medical Education Scale (GBMES). For Study 1 (*n* = 172), confirmatory factor analysis assessed the construct validity of the three-factor structure (awareness, beliefs, experience) and enabled deletion of redundant items. Study 2 (*n* = 457) tested the generalizability of the refined scale to a new sample. Combining Study 1 and 2, invariance testing for program of study and gender was explored. The relationship of the GBMES to demographic and gender politics variables was tested. The results were analyzed in R using confirmatory factor analysis and Multiple-Indicator-Multiple-Indicator-Cause models.

**Results:**

After analysis of the responses from the original 16-item GBMES (Study 1), a shortened measure of ten items fitted the data well (RMSEA = .063; CFI = .965; TLI = .951; Mean R-square of items = 58.6 %; reliability: .720–.910) and was found to generalize to a new sample in Study 2 (RMSEA = .068; CFI = .952; TLI = .933; Mean R-square of items = 55.9 %; reliability: .711–.892). The GBMES was found to be invariant across studies, gender, and program of study. Female students and those who supported gender equality had greater agreement for each of the factors. Likewise, postgraduate students reported higher scores on experience of gender bias than undergraduate students.

**Conclusion:**

The GBMES provides a validated short multidimensional measure for use in research and policy. Given its good reliability across different target populations and its concise length, the GBMES has much potential for application in research and education to assess students’ attitudes towards gender bias.

**Electronic supplementary material:**

The online version of this article (doi:10.1186/s12909-016-0774-2) contains supplementary material, which is available to authorized users.

## Background

Within the field of medicine, gender has been shown to be an ongoing factor contributing to health disparity [1–3]. Gender bias in medicine commonly occurs through the unequal treatment and diagnosis of a patient based on their sex and/or gender [4–6]. Stereotypical assumption about gender, as well as a lack of research and knowledge about sex-based differences, are also forms of gender bias that can negatively affect the medical diagnosis, treatment, and management of patients [7–10]. Numerous experts have argued that gender bias can be prevented in healthcare by incorporating gender issues into medical education [5, 6, 11]. However, in order to examine these issues, researchers and practitioners need access to an assessment tool that can be used to obtain quantifiable information on the extent and nature of students’ relationship with gender bias in medical education. To our knowledge there is currently no psychometrically validated, multidimensional scale that addresses this area of research interest. The closest measure is the Dutch Nijmegen Gender Awareness in Medicine Scale (N-GAMS) [12], which does not target issues related to medical education. To date, the most similar quantitative research is that of Morgan et al. [13] who included a few Likert and open response type items in their research on sexism in anatomy education. Their work was an important inspiration to the current research. However, given their purpose was not to develop a quantitative scale, this work did not hypothesize an underlying theoretical structure for their items, these items did not form a scale, and there was no evaluation of the psychometric properties of the items used. To address this need, a short multidimensional measure of gender bias was developed which could be used with medical students, and which covers participants’ awareness of gender bias, beliefs about how gender bias should be addressed, and experience of gender bias in a medical education context. We used Morgan et al.’s [13] items as inspiration and a starting point to develop a fully articulated multidimensional instrument. We suggest that research into gender bias in medical education must consider not only students *awareness* of gender bias, but also their *experiences* of gender bias, and their *beliefs* about how gender bias should be addressed.

Within this study, awareness is defined as a cognitive acknowledgement that gender bias exists; belief contains an attitudinal component that reflects a participant’s desire that gender bias be challenged or confronted and; experience includes being confronted with gender bias on either a first or second hand basis. Previous studies have aimed to measure student awareness of, or attitudes toward, gender in a medical context [12–14]. However, only one study [13] explored issues related to student’s belief about how gender issues should be addressed, while no study measured student’s direct and indirect experience of gender bias. While increased gender awareness among students has been shown to improve health equality in some instances [12], Hamberg [5] notes, “more knowledge does not eradicate the problem of knowledge-mediated bias or bias owing to notions and stereotyped ideas about men and women” (p. 241). Thus, students need to be aware not only of gender issues in their education, but also of the existence and outcomes of gender bias more broadly in medical practice. Further, an awareness of gender issues does not necessarily lead to a belief that action should be taken to prevent gender bias. Therefore, alongside ascertaining student beliefs about gender bias in medical education, it is important to also assess their beliefs about whether the risks of gender bias should be addressed in this context. Indeed, research has shown that the beliefs and attitudes of medical students, teachers, and practitioners can impact their behavior [15–18]. For this reason, attitudinal outcome measures are an important tool for examining medical student beliefs about if and how gender bias should be addressed during medical education. Lastly, while the effects of experiencing gender discrimination have been explored previously, these studies have focused on direct experiences of discrimination rather than on the broader experience of whether this exists in a medical education environment both directly and indirectly [19–21]. Importantly, research suggests that simply observing instances of discrimination can have a negative impact on psychological wellbeing, not only among marginalized groups, but also among non-target groups (e.g., negative effects on males observing sexism against females) [22]. Therefore, a general measure of experience of gender bias, covering both direct and indirect forms, is needed. Taken together, a measure of gender bias in medical education needs to address the degree to which students perceive medicine as male dominated, the degree to which they believe gender bias should be directly addressed in medical education, and their own experience of gender bias while undertaking medical coursework.

### Current study

Investigation into the existence and effects of gender bias is important for all facets of medical culture. Research with medical professionals and patients has begun to explore this issue and yet the significance of gender bias within medical education appears to be relatively underdeveloped. This is despite its critical role in the formation of attitudes and behaviors. Specifically, there is a paucity of measures that assess medical student reports of gender bias in their education, particularly ones that are multidimensional in nature. A lack of literature in this area highlights the need to explore medical students’ *awareness*, *beliefs,* and *experiences* of gender-related bias in their education. Correspondingly, this research aimed to develop and test a multidimensional measure designed to assess these three domains of awareness, belief, and experience in relation to gender bias. This measure was designed to be small and easily administered so that it could easily fit within larger research projects and be used by medical education programs. The aim of the study was to conduct a psychometric evaluation on a short, multidimensional measure of gender bias.

## Methods

The scale developed in this paper was designed for students studying anatomy as part of their university level education in medicine or allied health. The current research undertook a psychometric evaluation of the scale using a combination of exploratory and confirmatory factor analysis. The research consisted of one pilot study, then an initial validation (Study 1) and a replication study (Study2). The pilot, validation, and replication studies were all done with independent, cross-sectional samples of the same population of students in NSW Australia. The samples from Studies 1 and 2 were then combined to undertake invariance testing and to explore predictors of the scale factors. Invariance testing aimed to explore whether the scale operated in a similar manner for important groups (e.g., males and females).

A construct validity perspective was used by exploring both within- and between-network validity [23]. Within-network validity reflects the degree to which the hypothetical structure underlying the measure is reflected in the data collected. This was assessed with reference to the fit of the model (i.e., does the theoretical model fit the data), invariance tests (i.e., does the model act similarly across gender and program of study as expected), and exploration of the latent correlation matrix (i.e., is there sufficient evidence that the factors are measuring related but distinct factors as would be expected by a multi-dimensional instrument). Between-network validity considered the degree to which the factors in the scale are related to other factors in expected directions. Here we considered gender and gender politics.

### Participants

All data was collected from students studying anatomy at the University of Wollongong over a 2-year period, after ethics approval was obtained (University of Wollongong Human Research Ethics Committee, HE14/130). A pilot study to initially test and validate the measure was undertaken with a sample of 28 students from a third year undergraduate health science class in June 2014. The students completed the pilot instrument along with some demographic items during class time via pen-and-paper.

Two empirical studies (Studies 1 and 2) were then conducted with larger and more diverse populations that included both undergraduate health science students and postgraduate medical students to ensure that the measure was relevant and invariant across the entirety of medical and pre-medical education. Study 1 aimed to shorten the pilot instrument from 16 items to a target of 8 to 12 items, and consisted of a sample of 172 participants. Participants received an email link to the study via their university email. Study 1 targeted both undergraduate and post-graduate students. Participants completed the measure in their own time during September 2014.

The aim of Study 2 was to validate the short measure and consisted of a sample of 457 participants. Students enrolled in the first year anatomy course and in graduate-entry medicine at the University of Wollongong were invited to participate. Participants took part in the study during lab sessions from the period of April to May in 2015.

### Materials

The pilot study questionnaire was developed based, in part, on the work by Morgan et al. [13]. The items were used to explore the domain space and tapped content related to the factors defined in the literature review: awareness of gender bias in medical education, belief in the need to address gender bias in medical education, and the experience of gender bias during medical education. For each item, respondents selected a response from a 6-point Likert scale with options of strongly disagree (1), disagree (2), somewhat disagree (3), somewhat agree (4), agree (5), and strongly agree (6).

### Scale development

We requested access to the questionnaire developed by Morgan et al. [13] as it was the only previous study to explore gender bias in medical education from students’ perspectives. Within the questionnaire, six relevant themes were identified that were re-worked to facilitate a consistent attitudinal response format (i.e., agree-disagree Likert items; see below for details). Following the pilot study, item feedback from participants was provided. Students were instructed to highlight any words they did not understand and make notes on the page as to their thought processes while completing the items. The questions were then adjusted as a result. Finally, further items were developed in order to cover a greater breadth of the medical education domain space. As such, while the developed scale is clearly in line with many of the themes of the Morgan et al. study, all items is the scale were uniquely derived for this research.

### Data analysis

The factor structure of the full 16-item model was examined in Study 1 and then confirmed in Study 2 using confirmatory factor analysis. As a brief measure was desired, we followed the procedure outlined by Marsh et al. [23] for identifying candidate items for deletion. Specifically, candidate items for deletion were those with low loadings on the target factor, modification indices that suggested large loadings on non-target factors, and large residuals or modification indices that suggested correlated item residuals. The goal of using this procedure was to obtain an instrument that was short, provided a good fit to the data, and had factors that were distinguishable. Confirmatory factor analysis was also used in the two empirical studies to ensure construct validity and, via Study 2, to confirm that the shortened scale generalized to other samples. In all cases, data analysis was conducted in R [24] with major analysis undertaken using the lavaan package [25].

Following typical guidelines [26, 27], models were considered to fit the data well if: (a) the solution was well-defined, (b) parameter estimates were consistent with the theory proposed, and (c) the fit indices were acceptable, with an emphasis on those fit indices that did not favor small sample sizes. We thus report multiple indices in addition to the model chi-square because of its sensitivity to sample size. Based on commonly accepted criteria, Tucker-Lewis Index (TLI) ≥ .90, Comparative Fit Index (CFI) ≥ .90, and RMSEA < .08 were considered to provide evidence of model fit. Reliability of the factors was determined directly from the relevant CFA model using McDonald’s Omega^1^. Unlike Cronbach’s Alpha, Omega represents a true greatest lower bound on reliability [28].

In the final step of the analysis, we combined Studies 1 and 2 to explore invariance across study, gender, and program type (i.e. undergraduate or postgraduate). Invariance analysis fits and then compares measurement models in different sub-populations in order to ascertain whether the measurement structure is equivalent. That is, that the measure performs similarly in different groups [29, 30]. Evidence of invariance comes from comparing a well-fitting baseline model to alternate nested models. In such cases the sensitivity to the sample size of the chi-square does not merely relate to model fit but also to chi-square difference tests between nested models. Thus, we used the criteria proposed by Cheung and Resvold [31] and Chen [32] who suggested that invariance assumptions are supported when the difference between nested models corresponds to a ΔCFI ≤ .01 (we utilize the same criteria for the TLI) and a ΔRMSEA ≤ .015.

## Results

Demographic information and response rates for the pilot, initial validation (Study 1), and replication (Study 2) studies can be found in Table 1.

**Table 1:** Demographics

Demographics	Pilot	Study 1	Study 2
Sample size	28	172	457
Mean age in years (SD)	22(2.5)	25(2.5)	21(4.5)
Gender: Female	64 %	59 %	57 %
Program: Postgraduate	0 %	64 %	16 %
Response Rate	NA	52 %	80 %
Number of items tested	6	16	10

### Pilot study

Parallel analysis with exploratory factor analysis using data obtained from the pilot study was undertaken. This approach provided an analytical means (as opposed to a visual inspection of a scree plot of eigenvalues) of determining the appropriate number of factors in an exploratory factor analysis. In parallel analysis, eigenvalues from the observed data were compared to eigenvalues from random data of the same size. Factors that explained more variance than factors extracted from random data were retained [33]. For the pilot data, parallel analysis suggested three factors.

Based on an Oblimin (oblique) rotation, factor one accounted for 22 % of the variance in the items and related to awareness of gender bias in medical education (hereafter “awareness”; loadings = .44–1.00). Factor two accounted for 25 % of the variance in the items and related to whether participants believed medical education should explicitly address issues of gender bias (hereafter “belief”; loadings = .62–1.02). Lastly, factor three accounted for 25 % of the variance and related to whether participants themselves had experienced gender bias (hereafter “experience”; loadings = .57–1.01). Importantly, these results were consistent with the hypothesized factor structure and the correlation between factors was moderate ranging from *r* = .39 for awareness and belief to *r* = .12 for awareness and experience.

Following from this pilot study, the questionnaire was refined to more adequately cover the scope of the three factors. Six items were developed for gender bias awareness, and five items were developed for each of gender bias belief and experience, making a total of 16 items. This measure is referred to as the Gender Bias in Medical Education Scale (GBMES). More items were developed than needed with the goal of creating a short scale with a minimum of three and a maximum of four items per scale. This was done with the aim to develop a well-validated short form with clearly distinct factors that could easily fit into future studies without undue burden on participants (final items can be found in Table 2 and the item pool can be found in Additional file 1).

**Table 2:** Items and factor loadings for the 10-item Gender Bias in Medical Education Scale

Items	Scale	Loadings CFA Study 1	Loadings CFA Study 2
I believe that medicine is male dominated.	Awareness	.642	.568
Male bodies are treated as the default in medical education.	Awareness	.648	.688
In anatomy textbooks, reproductive chapters have more images of females than males.	Awareness	.571	.606
Medical studies are mainly done on males.	Awareness	.640	.599
I believe educators should raise awareness of the risks of gender bias in medicine.	Beliefs	.882	.872
I believe educators should raise awareness of the risks of gender bias in anatomical textbooks.	Beliefs	.909	.827
I believe anatomy educators should challenge gender-biased attitudes in the classroom.	Beliefs	.724	.695
I have seen evidence of gender bias in anatomy class activities.	Experience	.605	.604
I have encountered gender-biased *behaviors* among other students.	Experience	.967	.983
I have encountered gender-biased *attitudes* among other students.	Experience	.927	.898

*Notes. CFA* confirmatory factor analysis

### Study 1

Study 1 aimed to explore the initial factor structure and develop a short measure of the GBMES scale. Data consisted of a sample of 172 participants (59 % female; mean age of 25; 64 % of who were postgraduates). Submitting the hypothesized three-factor model to these data resulted in a poor fit (see Model 1, Table 3). Inspection of the factor loadings and modification indices suggested a number of poor loading items, redundant items (large correlated residual), and some items with moderate cross-loadings. Following the procedure for short form development by Marsh et al. [23], two items were removed from each of the scales. Following this, the revised 10-item measure (four awareness items, three belief items, and three experience items) was submitted to the data and displayed an excellent fit (Model 2, Table 3). Importantly, all factor loadings were above .50 and omega reliability estimates were acceptable for each of awareness (.720), belief (.879), and experience (.910). Furthermore, correlations between the three factors were moderate, indicating that they tapped related but different aspects of a common gender bias core. This reduced, 10-item measure represents the final scale (see Table 2 for items). However, it was possible that this reduced scale would not generalize to other samples, so this 10-item GBMES was therefore tested in a different student population.

**Table 3:** Confirmatory factor analysis for model fit for studies 1 and 2

	χ^2^	df	RMSEA	CFI	TLI
Study 1					
Model 1: 16 Item	271	101	.099	.858	.831
Model 2: 10 Item	54	32	.063	.965	.951
Study 2					
Model 3: 10 Item	99	32	.068	.952	.933
Study 1 and Study 2 combined					
Model 4: 10 Item	126	32	.069	.953	.934

*Notes. RMSEA* root mean square error of approximation, *CFI* comparative fit index, *TLI* Tucker Lewis index, *Df* degrees of freedom

### Study 2

Study 2 consisted of data from a sample of 457 medical students (57 % female; mean age of 21; all undergraduates) approximately 6 months after Study 1. The short form was fitted to these data using CFA, and displayed a good fit (Model 3, Table 3). Again, loadings were all above .50, correlations between factors were moderate, and omega reliability estimates were acceptable (awareness: .720, belief: .880, and experience: .910).

### Invariance tests across important groups

As a further test of the construct validity of the scale across studies, we combined the samples from Studies 1 and 2 to ensure adequate power and conducted invariance tests. Under the criteria for invariance noted above, there was evidence of configural (measurement structure equivalent across groups), weak (structure and factor loading equivalent across groups), strong (structure, factor loadings, and item intercept equivalent across groups), and strict (structure, factor loadings, item intercept, and item residuals equivalent across groups) factorial invariance across the two studies with little change in the fit indices. This suggested that the 10-item scale provided similar fit in both empirical studies. Evidence of invariance was likewise found for gender. For program of study results, configural weak, and strong invariance was supported but not strict invariance (Table 4). Taken together, there was evidence that the measurement structure of the 10-item GBMES operated in a similar manner across studies, genders, and programs of study when considering latent variables. However, for program of study the lack of invariant item residuals suggests that caution should be used when comparing across programs when using manifest variables [34].

**Table 4:** Latent correlation matrix for studies 1 and 2

	Awareness	Beliefs	Experiences
Study 1			
Awareness	1		
Beliefs	.561	1	
Experiences	.540	.413	1
Study 2			
Awareness	1		
Beliefs	.549	1	
Experiences	.378	.294	1

*Notes*. All correlations significant at *p* < .05

The consistent support for strong measurement invariance is an important requirement for comparing latent means as it provides evidence that such tests are comparing common measures (i.e., measures that are interpreted in a similar manner by both groups) [30]. On this basis we considered latent mean invariance. As can be seen in Table 4 there was a large change in fit indices when we constrained means to be equivalent across groups suggesting there were differences in latent means between Study 1 and 2, gender, and program of study (Table 5). As can be seen from Table 6, females had statistically significantly higher means than males for each of awareness, beliefs, and experience of gender bias. There were fewer differences by program of study, but postgraduate students did have statistically significantly higher means on experience of gender bias in their medical education compared with undergraduate students. Interestingly, there was evidence of differences in latent means between the two empirical studies, with participants from Study 1 displaying statistically significantly higher means on all three factors.

**Table 5:** Model fit: confirmatory factor analysis measurement invariance across studies, gender, and program of study

	χ^2^	df	RMSEA	CFI	TLI	Δχ^2^	Δdf	ΔRMSEA	ΔCFI
Study Invariance									
Configural	167	64	.072	.963	.948				
Weak: FL	172	71	.067	.964	.954	5	7	−.005	−.001
Strong: FL + I	200	78	.071	.956	.949	28*	14	.001	.007
Strict: FL + I + R^a^	209	88	.066	.957	.956	37*	23	.006	.006
Mean: FL + I + M	242	81	.080	.942	.936	75***	17	.008	.021
Gender Invariance									
Configural	158	64	.070	.963	.948				
Weak: FL	165	71	.066	.964	.954	7	7	−.004	−.001
Strong: FL + I	182	78	.066	.959	.953	24*	14	−.004	.004
Strict: FL + I + R^a^	195	88	.063	.958	.957	37*	23	−.007	.005
Mean: FL + I + M	213	81	.073	.949	.943	55***	17	.003	.014
Program of Study Invariance									
Configural	191	64	.080	.955	.937				
Weak: FL	198	71	.076	.955	.943	7	7	−.004	.000
Strong: FL + I^a^	238	78	.081	.944	.935	49**	14	.001	.011
Strict: FL + I + R	256	88	.078	.940	.939	65***	23	−.002	.015
Configural	282	81	.089	.929	.921	91***	7	.009	.026

*Notes*. Δ = Difference from configural model. ** *p* < .01; *** *p* < .001. ^a^ indicates best model. *FL* factor loadings, *I* item intercepts, *R* item residuals, *M* latent means

**Table 6:** Effect sizes for group difference in GBMES factors

	Awareness	Beliefs	Experience
Study (Group 2)	.224*	.458***	−.297**
Gender (Female)	.306***	.382***	.351***
Program (Postgraduate)	.009	−.016	.512***
Age^a^	−.091	−.055	.080
Gender Politics^a^	.258***	.366***	.127***

*Notes*. ^a^Estimates taken from MIMIC model. χ^2^ (46) = 163, RMSEA = .064, CFI = .949, TLI = .930. All other mean differences are taken from the relevant factor loading and intercept invariant model (strong invariance). * *p* < .05; ** *p* < .01; *** *p* < .001. Group in brackets indicates the direction of effects

### Predictors of GBMES

We were also interested in whether student responses on the GBMES differed by their age or their general agreement with gender politics. Gender politics was measured using a single item “I am supportive of gender equality” measured on the same 6-point Likert scale as the GBMES (where high scores demonstrated greater agreement). As these were ordinal variables, typical multi-group invariance models were not possible. Instead, a Multiple-Indicator-Multiple-Indicator-Cause model was run to test whether awareness, beliefs, or experience were predicted by either sympathy with gender politics or age. Results showed that age had no effect on any of the factors. Yet sympathy with gender politics had a significant relationship with all factors with greater agreement predicting higher scores on the awareness, belief, and experience scales.

## Discussion

The aim of the current study was to develop a short multidimensional measure of gender bias in medical education. As a result of a pilot study and two empirical studies, we developed a final 10-item measure named the Gender Bias in Medical Education Scale (GBMES) that showed good construct validity and reliability. Relationships between the scales and demographic variables were consistent with expectations. Similar to findings by Morgan et al. [13], females and those who supported gender equality were more likely to be aware of gender bias in medical education and to believe that gender bias should be addressed during education. However, the current study also found that females were more likely to report experiences of gender bias. This is consistent with a number of studies that have found that female medical students experience gender discrimination and sexism during their education and training [20, 35–38]. Again, those who reported greater sympathy with gender politics also reported a higher experience of gender bias. Likewise, students in postgraduate courses who thus had a longer tenure in medical education in general reported experiencing more gender bias. Importantly, age was not a significant predictor any of the factors and thus differences in program likely reflect time in medical education rather than natural development in political views over the lifetime. This likely explains the finding that participants from Study 1 displayed higher scores on all three factors since the majority of Study 1 participants were postgraduate students while Study 2 consisted mainly of undergraduate students in their first year of study.

This study revealed that a number of anatomy students were aware of gender bias in medical education, believed it should be addressed during education and had experienced gender bias themselves during their education. These results highlight the fact that medical education provides a unique opportunity to influence future healthcare providers by educating students on issues of gender and gender bias [5, 6, 11]. Beyond introducing gender issues into the medical curriculum, existing gender bias also needs to be eliminated from educational material. Gender bias has been demonstrated in medical textbooks [13, 39–41], medical curricula [42–44], and other educational tools and materials [4, 45], and exposure to gender bias has been shown to negatively influence an individual’s attitudes and decision-making [46–48]. Studies have also shown that medical educators often view gender issues as low priority topics in education [49–51]. The elimination of gender bias within medical education will provide students with fewer opportunities to adopt negative attitudes towards gender-related issues [5]. The GBMES is one way of monitoring students’ current perspectives of gender bias in medical education materials in order to ascertain when and where intervention would be best suited. Further, it can be used to highlight the importance of gender issues to medical educators and encourage them to prioritize it.

Importantly, while research has identified the potentially dangerous implications of gender bias for patients, a highly gender-biased medical culture has important implications for young physicians [19–21]. Research has shown that medical students can experience gender bias through discrimination and harassment [38, 52–54] and that these experiences often have an impact on their career opportunities and expectations [19–21, 52]. The GBMES provides a tool for much needed research on the extent to which students experience gender bias during medical education and the implications of this. Given the brevity of the scale, the GBMES can easily be incorporated into broader research projects providing greater scope to consider the many predictors and outcomes that gender bias in medical education may have. Likewise, the GBMES could be used to monitor programs designed to address gender bias in medical education.

### Limitations

The current research has many strengths; however, several limitations and avenues for future research need to be considered. First, while we considered the generalizability of the GBMES across gender and program of study, it should be noted that all three samples were taken from a single Australian university. As such it is critical that future research considers the degree to which the GBMES works with different samples from other institutions and countries. Second, the primary focus of the current research was on construct validity. While we did explore the effects of demographic data and sympathy with gender politics on the three factors of the GBMES, greater research on convergent and divergent validity are required. In particular, future research could consider the degree to which responses on the GBMES reflects general political beliefs and experiences versus those specific to the medical context. Finally, although three unique samples were used to ensure the scale replicated, no longitudinal data was collected and thus the stability of the measure over time has not been estimated.

## Conclusion

Analysis of two independent samples indicated that the GBMES provided a valid and reliable short multidimensional measure that was invariant across key demographics. The awareness, belief, and experience factors of the GBMES were distinct and related to gender, gender politics and years in education in expected directions. On this basis we suggest that the GBMES is an important tool for monitoring students’ awareness, beliefs, and experiences of gender bias during medical education and as a means of evaluating efforts to improve the representation of gender in medical education.

## Additional file

Additional file 1: Item Pool. This file contains a list of all questionnaire items that were used in the research. (DOCX 17 kb)

## Abbreviations

GBMES: Gender Bias in Medical Education Scale

## Footnotes

1 There are quite serious issues with alpha which suggests alternatives should be considered [55]. It has been shown that McDonald’s Omega gives a far better estimate of the lower bound of reliability and is a less biased estimate of the true reliability [28].

## References

[1] Baggio G, Corsini A, Floreani A, Giannini S, Zagonel V (2013). Gender medicine: a task for the third millennium. Clin Chem Lab Med.

[2] Bierman AS (2007). Sex matters: gender disparities in quality and outcomes of care. CMAJ.

[3] Kent JA, Patel V, Varela NA (2012). Gender disparities in health care. Mt Sinai J Med.

[4] Hamberg K, Larsson ML (2009). Still far to go – an investigation of gender perspective in written cases used at a Swedish medical school. Med Teach.

[5] Hamberg K (2008). Gender bias in medicine. Womens Health.

[6] Verdonk P, Benschop YWM, de Haes HCJM, Lagro-Janssen TLM (2009). From gender bias to gender awareness in medical education. Adv Health Sci Educ Theory Pract.

[7] Chiaramonte GR, Friend R (2006). Medical students’ and residents’ gender bias in the diagnosis, treatment, and interpretation of coronary heart disease’. Health Psychol.

[8] Hoyt MA, Rubin LR (2012). Gender representation of cancer patients in medical treatment and psychosocial survivorship research: changes over three decades. Cancer.

[9] Mosca L, Mochari H, Christian A, Berra K, Taubert K, Mills T, Burdick KA, Simpson SL (2006). National study of women’s awareness, preventive action, and barriers to cardiovascular health. Circulation.

[10] Smith EC. Gender-biased diagnosing, the consequences of psychosomatic misdiagnosis and ‘doing credibility’. Sociology Student Scholarship. 2011. http://scholar.oxy.edu/cgi/viewcontent.cgi?article=1005&context=sociology_student. Accessed 12 Jan 2016.

[11] Risberg G, Johansson E, Hamberg K (2009). A theoretical model for analysing gender bias in medicine. Int J Equity Health.

[12] Verdonk P, Benschop YWM, De Haes HCJM, Lagro-Janssen TLM (2008). Medical students’ gender awareness: construction of the Nijmegen gender awareness in medicine scale (N-GAMS). Sex Roles.

[13] Morgan S, Plaisant O, Lignier B, Moxham BJ (2013). Sexism and anatomy, as discerned in textbooks and as perceived by medical students at Cardiff University and University of Paris Descartes. J Anat.

[14] Andersson J, Verdonk P, Johansson EE, Lagro-Janssen T, Hamberg K (2012). Comparing gender awareness in Dutch and Swedish first-year medical students – results from a questionnaire. BMC Med Educ.

[15] Levinson W, Roter D (1995). Physicians’ psychosocial beliefs correlate with their patient communication skills. J Gen Intern Med.

[16] Rogers JC, Coutts L (2000). Do students’ attitudes during preclinical years predict their humanism as clerkship students?. Acad Med.

[17] van Ryn M, Burgess D, Malat J, Griffin J (2006). Physicians’ perceptions of patients’ social and behavioral characteristics and race disparities in treatment recommendations for men with coronary artery disease. Am J Public Health.

[18] Woloschuk W, Harasym P, Temple W (2004). Attitude change during medical school: a cohort study. Med Educ.

[19] Bartels C, Goetz S, Ward E, Carnes M (2008). Internal medicine residents’ perceived ability to direct patient care: impact of gender and experience. J Wom Health.

[20] Bruce A, Battista A, Plankey M, Johnson L, Marshall M (2015). Perceptions of gender-based discrimination during surgical training and practice. Med Educ.

[21] Stratton T, McLaughlin MA, Witte FM, Fosson SE, Nora LM (2005). Does students’ exposure to gender discrimination and sexual harassment in medical school affect specialty choice and residency program selection?. Acad Med.

[22] Swim JK, Hyers LL, Cohen LL, Ferguson MJ (2001). Everyday sexism: evidence for its incidence, nature, and psychological impact from three daily diary studies. J Soc Issues.

[23] Marsh HW, Ellis LA, Parada RH, Richards G, Heubeck BG (2005). A short version of the self description questionnaire II: operationalizing criteria for short-form evaluation with new applications of confirmatory factor analyses. Psychol Assess.

[24] R Core Team. R: a language and environment for statistical computing. R Foundation for Statistical Computing. 2014. <http://www.R-project.org/. Accessed 30 July 2015.

[25] Rosseel Y (2012). Lavaan: an r package for structural equation modeling. J Stat Softw.

[26] Marsh HW, Balla JR, McDonald RP (1988). Goodness of fit indexes in confirmatory factor analysis: the effect of sample size. Psychol Bull.

[27] McDonald RP, Marsh HW (1990). Choosing a multivariate model: Noncentrality and goodness of fit. Psychol Bull.

[28] Revelle W, Zinbarg RE (2009). Coefficients alpha, beta, omega and the glb: comments on Sijtsma. Psychometrika.

[29] Marsh HW, Parker PD, Morin A, Ntoumanis N, Myers ND (2016). Invariance testing across samples and time: cohort-sequence analysis of perceived body composition. An introduction to intermediate and advanced statistical analyses for sport and exercise scientists.

[30] Parker PD, Dowson M, McInerney D, McInnerney DM, Etten SV, Dowson M (2007). Standards for quantitative research in diverse sociocultural contexts. Standards in education.

[31] Cheung GW, Resvold RB (2002). Evaluating goodness-of-Fit indexes for testing measurement invariance. Struct Equ Model.

[32] Chen FF (2007). Sensitivity of goodness of fit indexes to lack of measurement invariance. Struct Equ Model.

[33] Floyd FJ, Widaman KF (1995). Factor analysis in the development and refinement of clinical assessment instruments. Psychol Assess.

[34] Marsh HW, Vallerand RJ, Lafrenière MAK, Parker P, Morin AJ, Carbonneau N (2013). Passion: does one scale fit all? Construct validity of two-factor passion scale and psychometric invariance over different activities and languages. Psychol Assess.

[35] Babaria P, Abedin S, Nunez-Smith M (2009). The effect of gender on the clinical clerkship experiences of female medical students: results from a qualitative study. Acad Med.

[36] Fnais N, Soobiah C, Hong Chen M, Lillie E, Perrier L, Tashkhandi M, et al. Harassment and discrimination in medical training: a systematic review and meta-analysis. Acad Med. 2014;89:817-27. 10.1097/ACM.0000000000000200 24667512

[37] Nora LM, McLaughlin MA, Fosson SE, Stratton TD, MurphySpencer A, Fincher RM, German DC, Seiden D, Witzke DB (2002). Gender discrimination and sexual harassment in medical education: perspectives gained by a 14-school study. Acad Med.

[38] Witte FM, Stratton TD, Nora LM (2006). Stories from the field: students’ descriptions of gender discrimination and sexual harassment during medical school. Acad Med.

[39] Dijkstra AF, Verdonk P, Lagro-Janssen AL (2008). Gender bias in medical textbooks examples from coronary heart disease, depression, alcohol abuse and pharmacology. Med Educ.

[40] Metoyer A, Rust R (2011). The egg, sperm, and beyond: gendered assumptions in gynecology textbooks. Womens Stud.

[41] O’Connell H, Sanjeevan K, Hutson J (2005). Anatomy of the clitoris. J Urol.

[42] Nicolette J, Jacobs MB (2000). Integration of women’s health into an internal medicine core curriculum for medical students. Acad Med.

[43] Henrich J, Viscoli C (2006). What do medical schools teach about women’s health and gender differences?. Acad Med.

[44] Verdonk P, Mans LJ, Lagro-Janssen TLM (2006). How is gender integrated in the curricula of Dutch medical schools? A quick-scan on gender issues as an instrument for change. Gend Educ.

[45] Verdonk P, Mans L, Lagro-Janssen A (2005). Integrating gender into a basic medical curriculum. Med Educ.

[46] Banaji MR, Hardin CD (1996). Automatic stereotyping. Psychol Sci.

[47] Davies PG, Spencer SJ, Steele CM (2005). Clearing the air: identity safety moderates the effects of stereotype threat on women’s leadership aspirations. J Pers Soc Psychol.

[48] Greenwald AG, Banaji MR (1995). Implicit social cognition: attitudes, self-esteem, and stereotypes. Psychol Rev.

[49] Risberg G, Johansson EE, Westman G, Hamberg K (2003). Gender in medicine – an issue for women only? A survey of physician teachers’ gender attitudes. Int J Equity Health.

[50] Risberg G, Hamberg K, Johansson EE (2003). Gender awareness among physicians – the effect of specialty and gender. A study of teachers at a Swedish medical school. BMC Med Educ.

[51] Risberg G, Johansson EE, Westman G, Hamberg K (2008). Attitudes toward and experiences of gender issues among physician teachers: a survey study conducted at a university teaching hospital in Sweden. BMC Med Educ.

[52] Hill E, Vaughan S (2013). The only girl in the room: how paradigmatic trajectories deter female students from surgical careers. Med Educ.

[53] Isaac C, Chertoff J, Lee B, Carnes M (2011). Do students’ and authors’ genders affect evaluations? A linguistic analysis of Medical Student Performance Evaluations. Acad Med.

[54] Johansson EE, Hamberg K (2006). From calling to a scheduled vocation: Swedish male and female students’ reflections on being a doctor. Med Teach.

[55] Sijtsma K (2009). On the use, the misuse, and the very limited usefulness of Cronbach’s alpha. Psychometrika.

