# Supplementary material for: A psychometric evaluation of the Gender Bias in Medical Education Scale
Source: BMC Med Educ. 2016 Sep 29;16:251. doi: 10.1186/s12909-016-0774-2 (PMC5041577; doi:10.1186/s12909-016-0774-2)
Supplement: Additional file 1: — Item Pool. This file contains a list of all questionnaire items that were used in the research. (DOCX 17 kb) [file 12909_2016_774_MOESM1_ESM.docx]

**Additional file**

Item Pool

| Items | Scale |
| --- | --- |
| **I believe that medicine is male dominated.** | Awareness |
| In textbooks, if I saw the female body was presented only to show how it differed from the male I would regard this as biased. | Awareness |
| **Male bodies are treated as the default in medical education.** | Awareness |
| **In anatomy textbooks, reproductive chapters have more images of females than males.** | Awareness |
| In anatomy textbooks, non-reproductive chapters have more images of females than males. | Awareness |
| **Medical studies are mainly done on males.** | Awareness |
| **I believe educators should raise awareness of the risks of gender bias in medicine.** | Beliefs |
| **I believe educators should raise awareness of the risks of gender bias in anatomical textbooks.** | Beliefs |
| **I believe anatomy educators should challenge gender-biased attitudes in the classroom.** | Beliefs |
| I believe anatomy educators should try to choose material that is not gender biased. | Beliefs |
| I believe anatomy textbooks should highlight when gender bias could impact on the understanding of anatomy. | Beliefs |
| I have seen gender biased behavior during my medical science education | Experience |
| **I have seen evidence of gender bias in anatomy class activities.** | Experience |
| **I have encountered gender-biased *behaviors* among other students.** | Experience |
| **I have encountered gender-biased *attitudes* among other students.** | Experience |
| I have seen evidence of gender bias in anatomy textbooks. | Experience |

*Notes.* Final items in bold
